# Potential Role of Certain Biomarkers Such as Vitamin B12, ROS, Albumin, as Early Predictors for Prognosis of COVID-19 Outcomes

**DOI:** 10.3390/medicines9060036

**Published:** 2022-06-15

**Authors:** Evgenia Lymperaki, Konstantina Kazeli, Georgia Variti, Magda Gerothanasi, Argyrios Gkinoudis, Ioannis Tsamesidis, Eleni Vagdatli

**Affiliations:** 1 Department of Biomedical Sciences, International Hellenic University, 57400 Thessaloniki, Greece; kkazeli@physics.auth.gr (K.K.); varitig@yahoo.gr (G.V.); magdagerothanasi@gmail.com (M.G.); johntsame@gmail.com (I.T.); vagdatli@mls.teithe.gr (E.V.); 2 Center for Interdisciplinary Research and Innovation (CIRI-AUTH), 57001 Thessaloniki, Greece; 3 School of Physics, Faculty of Sciences, Aristotle University of Thessaloniki, 54124 Thessaloniki, Greece; 4 Hippokration General Hospital of Thessaloniki, 54642 Thessaloniki, Greece; 5 School of Veterinary Medicine, Faculty of Health Sciences, Aristotle University of Thessaloniki, 54124 Thessaloniki, Greece; ginoudisa@gmail.com; 6 Department of Prosthodontics, School of Dentistry, Faculty of Health Sciences, Aristotle University of Thessaloniki, 54124 Thessaloniki, Greece

**Keywords:** COVID-19, ROS, vitamin B12, albumin, biomarkers

## Abstract

COVID-19 disease is still a major global concern because of its morbidity and its mortality in severe disease. Certain biomarkers including Reactive Oxygen Species (ROS), vitamins, and trace elements are known to play a crucial role in the pathophysiology of the disease. The aim of our study was to evaluate how certain biomarkers, such as ROS, biochemical indicators, trace elements in serum blood of 139 COVID-19 hospitalized patients, and 60 non-COVID cases according to age and sex variations, can serve as the predictors for prognosis of COVID-19 outcome. An attempt of correlating these biomarkers with the severity of the disease as well as with each other is represented. All subjects were hospitalized from April 2021 until June 2021. A statistically significant increase of B12 levels (*p* = 0.0029) and ROS levels (*p* < 0.0001) as well as a decrease in albumin and Total Protein (T.P.) levels (*p* < 0.001) was observed especially in the early stage of the disease before CRP and ferritin elevation. Additionally, a statistically significant increase in ferritin (*p* = 0.007), B12 (*p* = 0.035, sALT *p* = 0.069, Glucose *p* = 0.012 and urea *p* = 0.096 and a decrease in Ca *p* = 0.005, T.P *p* = 0.052 albumin *p* = 0.046 between stage B (CRP values 6–30 mg/L) and C (CRP values 30–100 mg/L) was evident. Thus, this study concludes that clinicians could successfully employ biomarkers such as vitamin B12, ROS and albumin as possible prognosis tools for an early diagnosis. In addition, the total biochemical profile can assist in the understanding of the severity of COVID-19 disease, and could potentially lead to a better diet or early pharmaceutical treatment to prevent some of the more acute symptoms.

## 1. Introduction

SARS-CoV-2 was first reported in Wuhan city, China, in the final week of December 2019. The affected organs varied to brain, central nervous system, lungs, heart, kidney, liver, pancreas, intestine, skin, and the blood vessels demonstrating multiple organ dysfunctions, and in more severe cases acute lung injury and respiratory distress syndrome, metabolic acidosis, heart failure, kidney injury, hypoxic encephalopathy, and eventually death of the illness [1,2]. From a pharmacological point of view, COVID-19 infection can be divided in three stages. In the early infection phase, prophylactic therapies dominate in order to assist the immune system, with possible antiviral treatment to patients whose immune system is very weak according to Center for Disease Control and Prevention (CDC). In the middle stage, the therapeutic regimen relies on monoclonal antibodies, nanobodies, and antiviral drugs to inhibit viral entry and replication. The final stage, which is the hyperinflammation phase, usually has the most severe symptoms, which can lead to multi-organ failure, and finally death. At that stage, treatment aims to suppress the immune response system, with certain drugs, monoclonal antibody, or other immunomodulatory agents, in order to halt the cytokine release syndrome/storm [3,4,5].

Early indicators as risk factors of severe illness can help to predict the severe progression of the disease in the early stages in terms of decreasing and controlling mortality. Recent research confirmed that some studied coronavirus cases have shown changes in the patients’ biochemical parameters and inflammatory markers, including lymphocyte count, neutrophil count, D-dimer status, C-reactive protein (CRP), erythrocyte sedimentation rate (ESR), and Interleukin-6. Other studies, likewise, demonstrated lymphocytopenia, high blood sugar, high gamma-glutamyl transferase (GGT), high lactate dehydrogenase (LDH), an increase in urea, cardiac troponin, creatine kinase, D-dimer, IL-6, and lower level of lactic acid levels and lymphocytes in more COVID-19 patients [6]. CRP and Lymphocyte ratio (LCR) are both identified as an inflammation marker that indicates the systemic inflammatory response, and can both be performed in nearly every laboratory [7,8]. In a multidisciplinary study, a new scoring system reported that the co-morbidity-age lymphocyte-LDH (CALL) score, could be a predictive value for COVID-19 progression with optimal sensitivity and specificity. Rodriguez et all showed that 2% of patients admitted to an Intensive Care Unit (ICU) were Vitamin B12 deficient [9]. Moreover, vitamin B12 supplementation has been showed to act favorably in chronic hepatitis C treatment despite the fact that B12 is metabolized in the liver [10].

Severe COVID-19 infection triggers imbalanced and uncontrolled cytokine response (called cytokine storm), exuberant endothelial inflammatory reactions, and vascular thrombosis. The pathological changes in organs and tissues are probably triggered by a host’s imbalanced reaction to the infection, e.g., excessive activation of immune and endothelial cells and platelets. Most likely, oxidative stress accompanying cell activation may profoundly impact COVID-19 pathogenesis [11]. Activity of the NOX isoforms is necessary for pathogen eradication, but excessive ROS production deteriorates the course of the disease. Increased concentration and activity of angiotensin II, resulting in oxidative stress and damage to cells’ macromolecules, can activate inflammatory cytokines, thus worsening the inflammatory response and contributing to the severity of the disease [12]. Studies have shown significantly lower levels of vitamin C in patients with COVID-19 associated with disease progression [13]. Serum vitamin D levels are reduced in these patients and could contribute to the severity of the disease. A research article studied the therapeutic effects of vitamin D supplementation in patients with COVID-19, and concluded that the supplement was associated with less severe disease progression and increased survival. The hormone melatonin acts as a free radical scavenger, protecting the integrity of the mitochondrial membrane during oxidative stress. In addition, it neutralizes the damage caused by viral infections, through interactions with a number of cellular proteins, signaling molecules and enzymes [14]. Natural antioxidants can compensate for the modified signaling pathways that are activated during the pathogenesis of COVID-19 [15]. The superoxide anion (O^2−^), hydroxyl radical (OH^−^), hydrogen peroxide (H_2_O_2_), hypochlorous acid (HOCl) and also NADPH oxidase-derived ROS are correlated with inflammation [16]. COVID-19 can cause DNA oxidation, lipid oxidation with elevated MDA levels, and protein oxidation. Consequently, COVID-19 is associated with increased production of all free radicals. Moreover, the high levels of angiotensin-2 during COVID-19 infection, increase the level of superoxide species.

Research gaps indicate that further research is needed to provide effective coping strategies to improve the severity of the disease and improve therapeutic outcomes, especially in patients with underlying complications [7].

Numerous previous studies suggested that serum albumin could be used as an early prognosis biomarker, by displaying that serum albumin levels were abnormally low in COVID-19 patients, and, thus, were associated with the severity of the disease. Therefore, dynamic monitoring of serum albumin is necessary and should be performed during treatment of patients with COVID, as a tool for assessing the prognosis of disease infections. A recent study showed that albumin binds to SARS-CoV-2 virus particles; this process can inhibit endothelial cap formation by inhibiting albumin binding sites. The authors suggested that albumin therapy should be urgently employed in patients with diffuse COVID-19 disease. A recent preliminary study showed that albumin administration reduced hypercoagulability. Overall, whether albumin administration to patients with COVID-19 is beneficial, is currently unknown. In addition to the therapeutic role of albumin, a number of studies have used albumin levels as a prognostic tool in patients with SARS-CoV-2 infection [17]. Consequently, studying the clinical and biochemical factors and characteristics between COVID-19 survivors and the deceased, may guide clinicians to identify severe cases at a very early stage of the disease and facilitate appropriate treatment. Based on the existing literature and being aware of the main biomarkers which were used to be tested when a COVID patient is admitted to the hospital, but especially based on the existing research differentiations on the role of biomarkers in the outcome of the disease, we proceeded to measure the aforementioned biomarkers for all systems such as renal, liver, heart, additional inflammatory biomarkers and ROS [7,18,19].

## 2. Materials and Methods

### 2.1. Study Population

In this study 199 subjects participated, 139 were COVID-19 diagnosed based on positive PCR test, and 60 were not positive diagnosed. All of them were hospitalized and none of them were vaccinated against SARS-CoV-2. Demographic data such as age, gender, BMI, and medical history were collected for all participants (Table 1).

### 2.2. Limitation of the Study

All the participants were selected based on their health status, the healthy subjects were considered to be those who had CRP levels <5 mg/L, excluding those with drug and supplement intake. No COVID-19 participants exhibited any other inflammatory disease by the time of hospital admission.

### 2.3. Samples Collection

Blood samples from 60 healthy patients (non-COVID-19) were collected, 24 male and 36 female, aged 9 to 89 years, who were examined or treated at the Hippocration General Hospital of Thessaloniki. Additionally, 139 blood samples of COVID-19 patients, 79 male and 60 female, aged 24–93 years old, who were hospitalized in the same hospital were collected.

All COVID-19 samples were classified into 4 groups according to the CRP value, and, therefore, according to the severity of COVID disease. The first group (Group A: *n* = 12, 5 male and 7 female) includes samples with CRP: <6 mg/L. The second group (Group B: *n* = 41, 18 male and 23 female) with CRP: 6–30 mg/L. The third group (Group C: *n* = 43, 26 male and 17 female) with CRP: 30–100 mg/L and finally the fourth group (Group D: *n* = 43, 30 male and 13 female) with CRP: >100 mg/L. The measured biochemical parameters in this research, as well as the method and analyzer used, are presented in Table 2.

All blood samples drawn from patients’ central line venous were immediately poured in tubes containing EDTA and centrifuged at 3000× *g* during 10 min in order to isolate plasma, and in tubes without EDTA to isolate serum for other serum-needed measurements. Plasma and serum samples were deeply frozen at −80 °C for ROS measurements, whereas all the other biomarkers were measured immediately. Blood determination of biomarkers, namely sALT, sAST, Glucose, Urea, Creatinine, Calcium, Phosphorus, Potassium, Sodium, Total Protein, Albumin, T. Cholesterol, Triglycerides and CRP concentration was performed on an ABBOT-Architect 16000 analyzer (Roche Diagnostics, Machelen, Belgium), B12 and Ferritin was determined by BECKMANCOULTER-DxI 800 analyzer (Roche Diagnostics, Machelen, Belgium), whereas, ROS concentration was measured using a Fluorometer TEKAN (Roche Diagnostics, Machelen, Belgium) with fluorometric method as described above. To determine the reactive oxygen species (ROS), the ROS-sensitive 2’,7’-dioxy-dichlorodihydrofluorescein (CM-H2DCFDA) primer fluorescent at 520 nm (lex = 480 nm) was used during oxidation. The oxidation of CM-H2DCFDA was measured fluorometrically on 96-well black wall microplates in TECAN fluorimeters. All measurements were performed for all biomarkers in the same sample the first day of hospitalization.

### 2.4. Ethical Consideration

The research quality control Committee of the General Hospital of Thessaloniki 2042021 approved the study; it was conducted in accordance with Good Clinical Practice guidelines and the Declaration of Helsinki. The confidentiality of participants was wholly preserved.

### 2.5. Statistical Analysis

To compare and correlate the ROS, Vitamin B12 and the other biomarker levels between the groups according to CRP value (Groups A–D), SPSS tool version 22.0 was utilized, in order to understand how the biomarkers can change the outcome of the disease. Descriptive statistics, presented as means ± standard deviations, were performed. Additionally, an inferential statistical analysis (*t*-test) was used to investigate the possible differences between the different groups. Pearson’s chi-square test or/and chi-square test of association was used to discover if there is a relationship between the categorized data, while Fisher’s exact test was used when expected variables were 2% of the total number of variables. In all statistical analyses, the level of significance (*p*-value) was set at α = 0.05.

## 3. Results

The average ROS levels and biochemical parameters of non-COVID-19 individuals and positive patients are presented in Table 3. In detail, as expected, CRP levels of COVID-19 patients appeared to be statistically significantly higher compared to the non-COVID-19 individuals (*p* < 0.0001). Further, statistically significant differences were observed between the two groups (COVID-19—non-COVID-19) regarding ferritin, B12, sALT, sAST, albumin, Urea, T. Cholesterol, and ROS levels (*p* < 0.001). However, albumin and total protein levels appeared to be lower for COVID-positive individuals. Our data presents a six times increased ferritin levels and two times increased B12 values for COVID-19 patients compared to the non-COVID-19 participants (*p* < 0.001). Regarding the liver function, sAST and sALT levels appeared in the borderline for the COVID patients and higher in comparison to the non-COVID-19 individuals. On the other hand, calcium and T. Cholesterol levels appeared statistically significant lower for COVID-19 patients (*p* < 0.001). The elected oxidative stress biomarker, ROS-H2DCFDA indicator revealed a high amount of ROS levels for COVID-19 patients compared to the non-COVID-19 individuals.

Once we found statistically significant differences in the biochemical biomarkers between the COVID-19 and non-COVID-19 participants, we elected to evaluate the group of COVID-19 patients based to the severity of the disease. We used CRP values to divide them in 4 groups as following: Group A: CRP < 6 mg/L, group B: CRP 6–30 mg/L, group C: CRP 30–100 mg/L, group D: CRP > 100 mg/L (Table 4a,b). The most significant result of our study appeared to be the gradual increase of ferritin levels based to the CRP values, from group A with 208.34 mg/L to group D with 1011.79 mg/L. Moreover, B12 levels appeared to drastically increase when CRP levels are more than 30 mg/L (*p* < 0.035). No statistically significant differences were observed concerning the liver function enzymes. An increase in glucose levels appeared statistically significant for group D in comparison to the other groups. Albumin levels appeared lower when CRP levels were increased dramatically for group D. Finally, ROS values were gradually increased from group A to group D (7633 to 16868 a.u) presenting a statistically significant increase especially for group A to B (*p* < 0.042).

ROS, B12, and CRP levels of non-COVID-19 and COVID-19 groups were significantly correlated with the tested biochemical biomarkers (Table 5). Regarding non-COVID-19 individuals, a weak correlation between ROS and biochemical markers was observed, such as for ferritin (r = 0.17), urea (r = 0.2), phosphorus (r = 0.2). However, an intermediate negative correlation between potassium and ROS levels was observed (r = −0.44). Stronger correlations are present between B12 levels and ferritin (r = 0.37), sAST (r = 0.26), sALT (r = 0.34), urea (r = 0.4), triglycerides (r = 0.23). An intermediate negative correlation appeared between albumin and B12 (r = −0.3). On the other hand, stronger correlations were observed for the COVID-19-positive group, indicating that the pathogenesis of COVID interfered dramatically with the inter-correlation of the biochemical parameters. Positive correlations between ROS levels and ferritin (r = 0.27), AST (r = 0.24), CRP (r = 0.32) were observed. Negative strong correlations were observed for ROS levels and albumin (r= −0.46), T. Cholesterol (r= −0.48). In addition, only a weak negative correlation was observed for albumin and B12 levels (r= −0.23). CRP levels revealed three important correlations with ROS (r = 0.32), Calcium (r = −0.29) and Ferritin (r = 0.41).

Regarding Table 6a–c, no statistically significant differences are observed between genders, for any biochemical markers, while also following the same trend in the four stages as in the total population. The only biomarker exhibiting a different trend is ferritin, whose levels are lower in women than in men in accordance with the literature, and is associated with the fact that women are less likely to develop a serious disease with severe symptoms.

## 4. Discussion

According to Table 1, it has been observed that only in Group A (COVID+ patients with mild symptoms) includes slightly younger patients comparatively to other groups, there is not great significant correlation with age to the severity of the disease. Additionally, other studies suggest that age could not be solely taken into account as a risk factor. Herein, is well known that none of the included biochemical markers are different among adults compared to age. When comparing females to males with COVID-19 (Table 1), females seem to exhibit a lower rate as the severity of the disease progresses (Group A (CRP < 6 mg/L) male 41.6%, female 58.4%), Group B (CRP < 6–30 mg/L) male 43.9%, female 56.1%, Group C (CRP < 30–100 mg/L) male 60.4%, female 39.6%, Group D (CRP > 100 mg/L) male 69.7%, female 30.3%), which is in accordance with other studies [20]. This fact is also confirmed by ferritin levels in the two genders (Table 6a,b). Evidence has shown that CRP is significantly associated with the severity and prognosis of excessive inflammatory responses, such as pneumonia resulting from a complication of COVID-19. CRP serves not only as a biomarker of inflammation, but also participates in its process. In recent studies, high CRP levels as well as age have been linked to severe forms of the disease [21,22]. Although the virological profile has not been fully elucidated as well as the involvement of CRP in its action, future studies will facilitate the prevention and treatment strategies against the disease. Ferritin is commonly known as an indicator of inflammatory conditions and infections. Numerous studies have shown that ferritin and CRP may potentially be markers for an early diagnosis of the systemic inflammatory response syndrome in cases of COVID-19 [23]. Recent research on ferritin has indicated that ferritin is elevated in cases of COVID-19 and may be associated with a worse clinical outcome [24,25], which comes in good agreement with this study. In this study there is also confirmed a statistically significant positive correlation between ferritin values and CRP values (r = 0.415). There is a gradual statistically significant increase in both CRP (*p* < 0.05) and ferritin values *p* < 0.05) in all groups (A, B, C, D). CRP and ferritin values can be used as biomarkers for the severity of the disease [26]. Moreover, lower female ferritin levels compared to male ferritin levels indicates that women are less likely to develop severe symptoms, giving a statistically better prognosis for the disease outcome. In addition, the heterogeneity of patients with COVID-19 infection suggests that other biomarkers except CRP and ferritin, could be used to evaluate various therapeutic methods, and improve the overall clinical progress. The reference value of B12 is 187–883 pg/mL, while, usually, laboratory tests indicate normal values range between 300–350 pg/mL. In our study, B12 average value in non-COVID-19 patients, was 334.8 pg/mL, including patients with some other infectious diseases, that could justify an increase in B12. In COVID-19 patients the average value of B12 was 501.85 pg/mL. In all groups (A, B, C, D), which have been separated according to CRP values and to the severity of the disease, B12 has an average range of 424 pg/mL. This may be due to a lack of metabolism in the liver, resulting in a blood level increase of cobalamin, a fact that is in accordance with recent research [27]. Former studies presented that supplementation of vitamin B12, vitamin D and magnesium could improve the disease progress [9]. Our study does not support the supplementation of Vitamin B12 after COVID-19 infection because the body will not be able to metabolize it. In those studies, the improvement in disease was maybe due to Vitamin D and magnesium supplementation. There is sufficient evidence that Vitamin B12 could be an early biomarker which can indicate a COVID-19 infection before CRP and ferritin increase [10].

According to recent studies, it can be assumed that an increase in oxidative stress levels could regress the severity of COVID-19 disease [28]. Additionally, in some studies focused on antioxidant levels (vitamin C, vitamin D and melatonin) in COVID-19 patients, it was demonstrated that these patients showed low values in antioxidant levels as well. Therefore, antioxidant supplements are recommended, which seems to result in increased survival rate [13,16]. ROS levels, according to our study, showed an increasing tendency depending on the severity of the disease. This is also confirmed by the average value of group D (16868) which tends to double compared to the average value of group A (8633). We also observe a statistically significant increase in ROS levels in group A, in the early stages of the disease progression. The value of significance that appears at the onset of the disease (*p* < 0.05) is also characteristically low. sAST was designated as a biochemical marker for the diagnosis of acute myocardial infarction in 1954. However, it has nowadays been replaced by cardiac troponins, and sAST and sALT increase from 15% to 53% in liver damage. According to the researchers, liver damage appears in patients with COVID-19, and it is much more common in those with a severe infection. A new study has found that the SARS-CoV-2 virus can be linked with the angiotensin-converting enzyme 2 (ACE2) in cholangiocytes, leading to cholangiocyte dysfunction and causing a systemic inflammatory response leading to liver damage [12]. Patients with severe COVID-19 appear to have higher rates of hepatic impairment [28]. In our study, sAST and sALT values showcased a statistically significant increase at the onset of the disease (*p* < 0.0001), and the most statistically significant trend is observed (*p* < 0.05) between group B and C in sAST values which could be used as a biomarker for the severity of the disease.

Research findings suggest that patients with severe COVID-19 symptoms typically have elevated blood glucose levels, which affect various biochemical pathways that facilitate SARS-CoV-2 infection [29,30,31]. In addition, it is assumed that diabetic patients have increased expression of angiotensin-converting enzyme-2 (ACE-2) receptors, making them vulnerable to SARS-CoV-2 infection. In our research, glucose rises (*p* = 0.089) in COVID-19-infected patients mainly in groups C and D (during disease progression) above normal values (*p* value between B–C = 0.012). This may be due to the disease itself, but also due to the administration of parenteral fluids that contain glucose.

According to studies, kidney failure is not uncommon in patients with COVID-19, even in those who did not have underlying kidney disease. Early reports found that up to 30% of patients treated with COVID-19 developed acute kidney disease [32,33,34,35,36]. In our data, urea is elevated in COVID+ patients (*p* = 0.005) while Creatinine, Sodium and Potassium levels remain within normal values. In some studies [37], hyperkaliemia and hyponatremia are common in symptomatic patients with COVID-19, who may sometimes present secondary clinical symptoms in this electrolyte imbalance. It is very important to determine the exact etiology of this electrolyte disorder, because the treatment differs depending on its pathophysiological mechanism [38]. Patients with hyponatremia and COVID-19 have a worse prognosis than those without electrolyte disturbances. Hypernatremia was less common in patients with COVID compared to those with hyponatremia (2.4% vs. 9.9%). Studies have shown that coronavirus disease affects kidney function by causing either hypo/hyperkalemia or hypo/hypernatremia [39]. Low levels of both trace elements appear to be more prevalent in COVID-19 cases and patients had higher hospital admission rates [40]. In our work, we do not observe a statistically significant difference in Potassium and Sodium between the four groups of COVID-19 patients and the values remain within normal limits.

Research findings suggest that Calcium and Phosphorus are a major boost against COVID-19. They are also useful biomarkers of clinical severity at the onset of symptoms. Low Calcium and Phosphorus more commonly result in severe or critical symptoms in patients with COVID-19 than in patients with moderate disease [41]. The findings of our work are similar, where Calcium shows a steady downward trend in COVID+ patients (*p* < 0.001), depending on the severity of the disease. The most statistically significant value *p* < 0.05 occurs in the middle of the disease progression and in the most severe cases. Phosphorus shows a statistically significant decrease after the initial stage (*p* < 0.05) when the infection begins to become more apparent. The available literature also shows that hypoalbuminemia in patients with COVID-19 is associated with an excessive increase in inflammatory cytokines, coagulation parameters as well as the progression to more severe stages of the disease involving an increased need for hospitalization [17]. Thus, in our study, total albumin as well as albumin show a downward course (*p* < 0.001), from the onset of the disease and throughout its duration, depending on its severity. There is also a statistically significant decrease of albumin and Total Protein levels from the early stages of the disease (*p* < 0.01) and, thus, these values could also serve as early biomarkers of COVID-19 infection. Dynamic monitoring of serum albumin is therefore necessary and should be performed during treatment of patients with COVID-19 as a tool to assess the prognosis of infection. Research has shown that low T. Cholesterol and high triglyceride concentrations measured during hospitalization are strong predictors of a serious course of the disease. Taking into account numerous studies which have observed the decreasing tendency of the total, LDL and HDL Cholesterol levels, the lipid profile should be considered a sensitive early predictor of the COVID-19 outcome disease along with the common inflammatory markers (e.g., CRP, ESR) and should be measured in patients with COVID-19 [42,43,44,45,46,47]. In our research all COVID+ patients show lower T. Cholesterol (*p* = 0.005) and higher triglycerides values (*p* = 0.05). This may be due to their hospitalization and perhaps due to lack of nutrition.

## 5. Conclusions

Many biochemical markers, including ferritin, B12, and ROS, could serve as COVID-19 disease severity markers. Specifically, B12, ROS, T.P and Albumin could play from the very early stages of the disease an important role as biomarkers as early indicators of the infection; while, other biochemical markers, such as ferritin and CRP increase as the disease progresses. In some diagnostic biomarkers (ferritin, B12, sAST, glucose, urea, Ca, T.P., albumin, triglycerides) there is a statistically significant increase between groups B and C when the disease begins to settle in its most severe form. Additionally, it is of utmost importance to always keep in mind that the therapeutic regiment to be followed for the patients’ treatment should not be solely based on numbers but also on diagnostic and clinical observations, as well. Our study suggests that B12 and ROS can be used as potential early biomarkers for the progression of COVID-19 disease.

The symptoms of COVID-19 differ and depend on the patient’s health background, as well as their genetic profile. Therefore, it is necessary to evaluate all prognostic tools to prevent the severe and critical outcome of the disease, and to monitor all biochemical and inflammatory biomarkers at all stages of COVID-19.

## Figures and Tables

**Table 1:** Characteristics of patients Age, Male/female, Severity of the disease.

	Non COVID	COVID			
	Healthy (CRP < 5 mg/L)	Group A (CRP < 6 mg/L)	Group B (CRP: 6–30 mg/L)	Group C (CRP: 30–100 mg/L)	Group D (CRP > 100 mg/L)
Male	24	5 (41.6%)	18 (43.9%)	26 (60.4%)	30 (69.7%)
Female	36	7 (58.4%)	23 (56.1%)	17 (39.6%)	13 (30.3%)
Age	9–89	29–78	28–93	33–90	24–91
Total participants	60	12	41	43	43
BMI (kg/m^2^)	30.1	29.5	30.3	30.4	31.1

**Table 2:** Methods and analyzer used to measure the biochemical parameters.

Biochemical Test	Method	Analyzers
CRP	Latex indirect agglutination test/or turbidimetric	ABBOT-Architect 16000
B12	Chemiluminescent microparticle Immunoassay	BECKMANCOULTER-DxI 800
Ferritin	Chemiluminescent microparticle Immunoassay	BECKMANCOULTER-DxI 800
sALT	Kinetic colorimetric by IFCC	ABBOT-Architect 16000
sAST	Kinetic colorimetric by IFCC	ABBOT-Architect 16000
Glucose	Enzymatic, Colorimetric/Hexokinase (HΚ/G-6-PDH)	ABBOT-Architect 16000
Urea	Enzymatic, Colorimetric /Urease/GLDH	ABBOT-Architect 16000
Creatinine	Sodium Picrate, Colorimetric, kinetic (Jaffe)	ABBOT-Architect 16000
Calcium	Arsenazo ΙΙΙ	ABBOT-Architect 16000
Phosphorus	Phosphomolybdate UV	ABBOT-Architect 16000
Potassium	Potentiometric (ISE direct)	ABBOT-Architect 16000
Sodium	Potentiometric (ISE direct)	ABBOT-Architect 16000
Total Protein	Βiuret method, colorimetric	ABBOT-Architect 16000
Albumin	Green of Bromocresol/BCG, colorimetric	ABBOT-Architect 16000
T. Cholesterol	Cholesterol oxidase (CHOD/POD)	ABBOT-Architect 16000
Triglycerides	Enzymatic, colorimetric (GPO/POD)	ABBOT-Architect 16000
ROS	Fluorometric	Fluorometer TEKAN

**Table 3:** Βiochemical parameters between confirmed and suspected patients at the time of admission.

Parameters	Normal Values	Average Values COVID −	Average Values COVID +	*p* Values
CRP	<6 mg/L	1.7	80.47	<0.0001
Ferritin	10–290 mg/L	137	663.82	<0.0001
B12	187–883 pg/mL	334	501.85	0.0029
sAST	5–35 U/L	25	40.73	0.0007
sALT	5–35 U/L	27	43.51	0.0005
Glucose	55–110 mg/dL	110	124.68	0.089
Urea	15–43 mg/dL	40	53.31	0.005
Creatinine	0.5–1.1 mg/dL	1.15	1.22	0.35
Ca	8.8–10.6 mg/dL	9.21	8.19	<0.0001
P	2.5–4.5 mg/dL	3.9	3.67	0.13
Κ	3.5–5.1 mmol/L	4.67	4.36	0.014
Νa	136–145 mmol/L	140	140.02	0.385
Τ.P	6.0–8.3 g/dL	7	6.1	<0.0001
Albumin	3.4–5.4 g/dL	4.2	3.39	<0.0001
T. Cholesterol	<200 mg/dL	183	151.61	0.0005
Triglycerides	40–140 mg/dL	115	140.43	0.05
ROS	(A.U)	8602	14,201	<0.0001

**Table 4:** (a) Biochemical parameters according to CRP values; (b) *p*- value between groups.

**(a)**					
**Biochemical Parameters**	**Normal Values**	**Av. Values Group A (CRP < 6 mg/L)**	**Av. Value Group Β (CRP 6–30 mg/L)**	**Av. Value Group C (CRP 30–100 mg/L)**	**Av. Value Group D (CRP >100 mg/L)**
CRP	<6 mg/L	2.45	20.38	57.8	182.2
Ferritin	10–290 mg/L	208.34	385.02	716.88	1011.79
B12	187–883 pg/mL	431.83	424.58	593.16	503.76
sAST	5–35 U/L	25.58	32.56	45.46	48.02
sALT	5–35 U/L	48	38.09	42.53	48.41
Glucose	55–110 mg/dL	100.91	105.36	137.27	137.13
Urea	15–43 mg/dL	53.91	45.58	54.65	59.18
Creatinine	0.5–1.1 mg/dL	1.43	1.07	1.113	1.42
Ca	8.8–10.6 mg/dL	8.75	8.47	8.05	7.91
P	2.5–4.5 mg/dL	4.288	3.42	3.57	3.84
Κ	3.5–5.1 mmol/L	4.283	4.35	4.39	4.37
Νa	136–145 mmol/L	139.08	139.8	140.04	140.48
Τ.P	6.0–8.3 g/dL	6.66	6.16	5.89	6.08
Albumin	3.4–5.4 g/dL	3.9	3.43	3.27	3.35
T. Cholesterol	<200 mg/dL	149	139	164.54	144.45
Triglycerides	40–140 mg/dL	112.4	98	163.72	144.91
ROS	(A.U)	7634	13929	13380	16868
**(b)**					
**Biochemical Parameters**		***p* Value A–Β**		***p* Value B–C**	***p* Value C–D**
Ferritin		0.134		0.007	0.069
B12		0.478		0.035	0.178
sAST		0.121		0.069	0.389
sALT		0.189		0.294	0.232
Glucose		0.357		0.012	0.496
Urea		0.165		0.096	0.271
Creatinine		0.152		0.378	0.077
Ca		0.151		0.005	0.197
P		0.024		0.253	0.189
Κ		0.354		0.362	0.423
Νa		0.203		0.371	0.312
Τ.P		0.015		0.052	0.215
Albumin		0.001		0.046	0.215
T. Cholesterol		0.231		0.120	0.105
Triglycerides		0.347		0.071	0.269
ROS		0.042		0.402	0.060

**Table 5:** Correlation of B12, ROS and CRP values with the analyzed biochemical parameters.

Test	Non-COVID		COVID		
	ROS (r +/−)	B12 (r +/−)	ROS (r +/−)	B12 (r +/−)	CRP (r +/−)
Ferritin	0.168	0.377	0.272	0.067	0.415
Β12	−0.004	1.000	0.062	1.000	0.038
sAST	0.054	0.266	0.241	0.131	0.197
sALT	0.037	0.340	0.031	0.136	0.074
Glucose	0.021	0.166	−0.028	−0.0004	0.112
Urea	0.206	0.396	0.166	0.086	0.130
Creatinine	0.157	0.048	−0.008	0.111	0.050
CRP	0.039	0.105	0.324	0.038	1.000
Ca	−0.0001	0.038	−0.279	−0.166	−0.299
P	0.202	0.028	0.054	0.131	0.058
Potassium	−0.441	−0.040	−0.028	−0.110	0.056
Sodium	−0.044	0.007	0.203	0.123	0.145
T.P	0.019	−0.173	−0.307	−0.082	−0.154
Albumin	−0.157	−0.302	−0.457	−0.230	−0.169
T. Cholesterol	0.043	−0.054	−0.480	−0.070	−0.193
Triglycerides	0.115	0.229	−0.116	−0.185	−0.002
ROS		−0.004		0.062	0.324

**Table 6:** (a): Biochemical parameters according to CRP values in females; (b) biochemical parameters according to CRP values in males; (c) biochemical parameters in COVID-participants according to gender.

**(a)**					
**Biochemical Parameters**	**Normal Values**	**Av. Value Group A (CRP < 6 mg/L)**	**Av. Value Group Β (CRP 6–30 mg/L)**	**Av. Value Group C (CRP 30–100 mg/L)**	**Av. Value Group D (CRP >100 mg/L)**
CRP	<6 mg/L	2.0	22.7	53.4	162.2
Ferritin	10–290 mg/L	111.6	258.4	522.5	634.2
B12	187–883 pg/mL	573.4	418.2	716.7	583.2
sAST	5–35 U/L	18.1	31.8	40.2	47.0
sALT	5–35 U/L	35.0	34.2	35.5	49.2
Glucose	55–110 mg/dL	93.0	101.39	124.0	145.5
Urea	15–43 mg/dL	56.7	42.1	45.5	53.7
Creatinine	0.5–1.1 mg/dL	1.7	1.1	0.9	1.4
Ca	8.8–10.6 mg/dL	8.8	8.5	8.0	8.2
P	2.5–4.5 mg/dL	4.9	3.3	3.8	3.9
Κ	3.5–5.1 mmol/L	4.2	4.2	4.0	4.2
Νa	136–145 mmol/L	138.4	139.2	141.1	140.3
Τ.P	6.0–8.3 g/dL	6.7	6.0	5.8	6.5
Albumin	3.4–5.4 g/dL	3.9	3.4	3.2	3.4
T. Cholesterol	<200 mg/dL	149.0	142.5	167.0	126.5
Triglycerides	40–140 mg/dL	112.4	117.0	162.3	108.0
ROS	(A.U)	5380.0	14,082.7	13,373.6	17,067.8
**(b)**					
CRP	<6 mg/L	3.1	17.3	60.7	190.9
Ferritin	10–290 mg/L	343.6	546.7	844.0	1181.1
B12	187–883 pg/mL	233.6	432.7	512.4	469.4
sAST	5–35 U/L	36.0	33.5	48.8	48.5
sALT	5–35 U/L	66.2	43.1	47.1	48.0
Glucose	55–110 mg/dL	112.0	110.4	145.9	133.5
Urea	15–43 mg/dL	50.0	50.0	60.7	61.5
Creatinine	0.5–1.1 mg/dL	0.9	1.1	1.2	1.5
Ca	8.8–10.6 mg/dL	8.6	8.4	8.1	7.8
P	2.5–4.5 mg/dL	3.5	3.6	3.4	3.7
Κ	3.5–5.1 mmol/L	4.3	4.6	4.6	4.4
Νa	136–145 mmol/L	140.0	140.6	139.3	140.6
Τ.P	6.0–8.3 g/dL	6.5	6.3	5.6	6.1
Albumin	3.4–5.4 g/dL	3.8	3.5	3.3	3.3
T. Cholesterol	<200 mg/dL	141.0	135.5	163.6	148.4
Triglycerides	40–140 mg/dL	101.0	79.0	164.3	157.2
ROS	(A.U)	10,701.0	13,698.1	13,384.9	16,831.4
**(c)**					
**Parameters**	**Normal Values**	**Average Values COVID-MALE**		**Average Values COVID-FEMALE**	
CRP	<6 mg/L	1.4		1.9	
Ferritin	10–290 mg/L	144.6		132.4	
B12	187–883 pg/mL	357.9		319.4	
sAST	5–35 U/L	24.3		25.6	
sALT	5–35 U/L	27.7		26.8	
Glucose	55–110 mg/dL	106.0		112.9	
Urea	15–43 mg/dL	40.2		40.5	
Creatinine	0.5–1.1 mg/dL	1.0		1.2	
Ca	8.8–10.6 mg/dL	9.2		9.2	
P	2.5–4.5 mg/dL	3.6		4.1	
Κ	3.5–5.1 mmol/L	4.9		4.6	
Νa	136–145 mmol/L	140.8		139.8	
Τ.P	6.0–8.3 g/dL	7.1		6.9	
Albumin	3.4–5.4 g/dL	4.3		4.1	
T. Cholesterol	<200 mg/dL	158.7		199.4	
Triglycerides	40–140 mg/dL	112.1		116.3	
ROS	(A.U)	8337.5		8778.6	

## Abbreviations

ALT: Alanine aminotransferase, AST: Aspartate aminotransferase, CRP: C-Reactive Protein, ROS: Reactive Oxygen Species, Na: Sodium, Ca: Calcium, K: Potassium, P: Phosphorus, T.P.: Total Protein, COVID-19: 2019 novel coronavirus disease, SARS-CoV-2: Severe acute respiratory syndrome coronavirus 2
